# The Role of Auxiliary Subunits for the Functional Diversity of Voltage-Gated Calcium Channels

**DOI:** 10.1002/jcp.24998

**Published:** 2015-05-26

**Authors:** Marta Campiglio, Bernhard E Flucher

**Affiliations:** Division of Physiology, Department of Physiology and Medical Physics, Medical University InnsbruckInnsbruck, Austria

## Abstract

Voltage-gated calcium channels (VGCCs) represent the sole mechanism to convert membrane depolarization into cellular functions like secretion, contraction, or gene regulation. VGCCs consist of a pore-forming α_1_ subunit and several auxiliary channel subunits. These subunits come in multiple isoforms and splice-variants giving rise to a stunning molecular diversity of possible subunit combinations. It is generally believed that specific auxiliary subunits differentially regulate the channels and thereby contribute to the great functional diversity of VGCCs. If auxiliary subunits can associate and dissociate from pre-existing channel complexes, this would allow dynamic regulation of channel properties. However, most auxiliary subunits modulate current properties very similarly, and proof that any cellular calcium channel function is indeed modulated by the physiological exchange of auxiliary subunits is still lacking. In this review we summarize available information supporting a differential modulation of calcium channel functions by exchange of auxiliary subunits, as well as experimental evidence in support of alternative functions of the auxiliary subunits. At the heart of the discussion is the concept that, in their native environment, VGCCs function in the context of macromolecular signaling complexes and that the auxiliary subunits help to orchestrate the diverse protein–protein interactions found in these calcium channel signalosomes. Thus, in addition to a putative differential modulation of current properties, differential subcellular targeting properties and differential protein–protein interactions of the auxiliary subunits may explain the need for their vast molecular diversity. *J. Cell. Physiol. 999: 00–00, 2015*. © 2015 The Authors. *Journal of Cellular Physiology* Published by Wiley Periodicals, Inc. J. Cell. Physiol. 230: 2019–2031, 2015. © 2015 Wiley Periodicals, Inc.

Voltage-gated calcium channels (VGCCs or Ca_V_) are unique among ion channels in that they combine two distinct signaling functions. First, like other voltage-gated ion channels, they open in response to changes in membrane potential allowing the influx of calcium ions down a steep electro-chemical gradient (Fig. 1A). This cation current further depolarizes the cell and thus plays important roles in the generation and shaping of action potentials, like in cardiac myocytes and in many neurons. Second, because the calcium ion is one of the cell’s principal second messengers, VGCCs also function as a major activator of calcium-mediated cellular functions. Due to this dual role VGCCs are in the unique position to transduce membrane depolarization into cell functions like excitation–contraction coupling in muscle, excitation–secretion coupling in hormone-secreting and nerve cells, and activity-dependent gene regulation in a host of excitable cells. Consequently, dysfunctions of VGCCs due to disease or genetic aberrations result in a wide spectrum of disorders including developmental disabilities, neurological, and cardio-vascular diseases (Cain and Snutch, 2011; Simms and Zamponi, 2014). Accordingly VGCCs serve as potent drug targets in the therapy of many of these diseases (Bauer et al., 2010; Striessnig et al., 2014).

**Figure 1:**
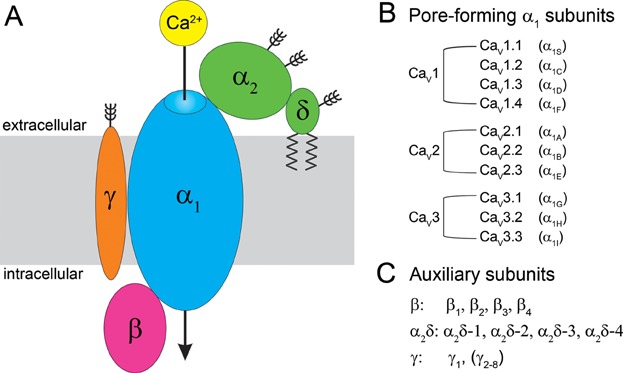
Molecular organization and genetic diversity of voltage-gated calcium channels. A: Subunit composition of VGCCs. Auxiliary β and α_2_δ subunits interact with Ca_V_1 and Ca_V_2 channels, while γ subunits associate to VGCC complexes only in muscle. B: List of the calcium channels genes and of the corresponding α_1_ subunits. C: List of auxiliary VGCC subunits genes.

The great diversity of VGCC-mediated cell functions is mirrored by a considerable molecular diversity of the VGCC family. In mammals the principal channel protein, called the α_1_ subunit, is encoded by ten genes subdivided in three channel classes which differ with respect to current properties and pharmacology: L-type Ca_V_1.1-4 and non-L-type Ca_V_2.1-3 together representing the high-voltage activated channels, and Ca_V_3.1-3 the class of low-voltage activated channels (Catterall, 2011) (Fig. 1B). The α_1_ subunits contain the voltage-sensing domains and the conduction pore with the selectivity filter and the activation gate. In addition, the α_1_ subunits contain important modulatory sites, protein–protein interaction domains, as well as the major drug binding sites (Fig. 2). Expression of an α_1_ subunit is sufficient to reconstitute functional channels in heterologous cells, albeit with reduced current densities and gating properties distinct from those of the native channels. Current densities of Ca_V_1 and Ca_V_2 channels are enhanced and gating properties normalized by the co-expression of auxiliary channel subunits, giving rise to the concept that auxiliary subunit modulate channel properties. The classical auxiliary subunits include the cytoplasmic β subunits (β_1_, β_2_, β_3_, β_4_), the extracellular α_2_δ subunit (α_2_δ-1, -2, -3, -4), and in skeletal muscle an integral membrane protein, the γ_1_ subunit (Fig. 1C). Multiple splice variants of each Ca_V_ subunit isoform further add to the molecular diversity of the VGCCs (Catterall, 2011).

**Figure 2:**
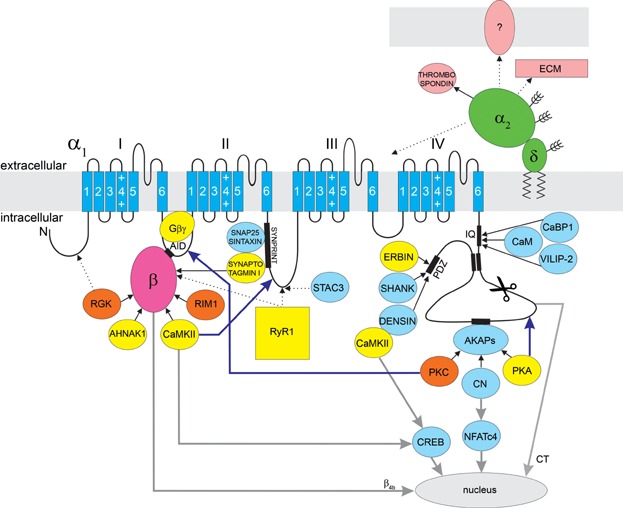
Schematic representation of the predicted membrane topology of the α_1_ subunit and of its interaction partners. In yellow are interaction partners that can interact only with specific β isoforms, or the effects of which are β isoform specific. In orange are interacting proteins that require any β subunit bound to the AID in order to exert an effect on the VGCC complex. Light blue indicates the interaction partners for which no β effect has been reported. In pink are extracellular interaction partners of α_2_δ. Continuous black arrows indicate verified interactions, whereas dotted arrows indicate suggested interactions. Blue arrows indicate up-stream modulation of the channel, while grey arrows indicate down-stream signaling to the nucleus. RGK proteins and RIM1 bind to all β isoforms. For RGK proteins alternative binding sites have been suggested (e.g., on the N-terminus). PKC requires a β subunit for the enhancement of Ca_V_2.2 and Ca_V_2.3 currents. Synaptotagmin I, Ahnak1, RyR1, and CaMKII bind specific β isoforms. Erbin facilitates Ca_V_1.3 currents only in the presence of specific β isoforms, while PKA modulates Ca_V_1 currents with different strength depending on the associated β isoform. G_βγ_ proteins require a β subunit bound to exert current inhibition, whereby the β isoform determines the strength of inhibition (see Table1 for details). The β_4b_ isoform and a C-terminal fragment of Ca_V_1.2 and Ca_V_2.1 (CT) can also translocate to the nucleus, where they regulate transcription. Except for thrombospondin, no interaction partners of α_2_δ subunits have been described. The majority of α_2_δ is extracellular, ideally positioned to interact with the extracellular matrix (ECM) and for possible involvement in cell–cell contacts.

Although the minimal subunit composition of α_1_/α_2_δ/β defines functional high-voltage activated calcium channels, in their native environment they typically function in the context of macromolecular signaling complexes, including various up-stream modulators, down-stream effectors, as well as adapter and scaffold proteins. Growing evidence indicates that the β subunits, and perhaps also the α_2_δ subunits, play important roles in orchestrating these functional calcium channel signaling complexes. Moreover, a number of up- and down-stream signaling processes require the presence of specific auxiliary subunit isoforms, indicating the importance of the large molecular diversity of calcium channel subunits beyond differential modulation of VGCC current properties.

## Auxiliary Calcium Channel Subunits

The molecular diversity of VGCCs is greatly enhanced by the auxiliary calcium channel subunits, which promote membrane expression and modulate the current properties. The original purification of VGCCs from skeletal muscle revealed that calcium channels are protein complexes consisting of the aforementioned four non-covalently associated subunits (Fig. 1A and C): α_1_, β, α_2_δ, and γ (Curtis and Catterall, 1984; Takahashi et al., 1987).

The α_2_δ subunits are highly glycosylated extracellular proteins encoded by four genes in mammals (Dolphin, 2013; Geisler et al., 2014). The gene product is post-translationally cleaved into the α_2_ and the δ polypeptides which remain associated with each other by disulfide bonds (Ellis et al., 1988; Calderon-Rivera et al., 2012). Initially the δ subunit was proposed to form a transmembrane helix with the short C-terminus exposed to the cytoplasmic compartment (Ellis et al., 1988). More recent evidence suggests that α_2_δ is anchored to the membrane by a GPI-anchor at the C-terminus of the δ polypeptide (Davies et al., 2010). Due to their large and highly glycosylated extracellular structure α_2_δ subunits are ideally situated to interact with components of the extracellular matrix (Geisler et al., 2014). Furthermore, α_2_δ-1 and -2 contain a binding site for gabapentin and pregabalin, two drugs used to treat epilepsy and neuropathic pain (Dolphin, 2013). The nature of α_2_δ binding and interaction with the α_1_ subunit is poorly understood, but co-expression of an α_2_δ subunit with an α_1_ subunit in heterologous cells consistently augmented the expression of functional channels (Dolphin, 2013). Knockdown or knockout of α_2_δ in muscle cells results in a loss of the specific activation kinetics of the skeletal and cardiac muscle Ca_V_1 isoforms (Sipos et al., 2000; Obermair et al., 2005; Tuluc et al., 2007; Obermair et al., 2008; Fuller-Bicer et al., 2009). Therefore, α_2_δ appears to function in both targeting and/or stabilization of the channel in the membrane and in shaping the specific gating properties of different channel isoforms.

The calcium channel β subunit is a cytoplasmic protein that binds to Ca_V_1 and Ca_V_2 channels at a high-affinity binding site (AID, for α interaction domain) in the cytoplasmic loop connecting repeats I and II of the α_1_ subunit (Pragnell et al., 1994). Mammals have four β subunit genes. The domain structure of β subunits bears resemblance to that of membrane-associated guanylate kinase proteins (MAGUKs), containing a conserved SH3 domain and a guanylate kinase (GK) domain (reviewed in Buraei and Yang (2010)). In the β subunit the GK domain lacks kinase activity but forms the binding pocket for the AID domain of the α_1_ subunits (Chen et al., 2004; Opatowsky et al., 2004; Van Petegem et al., 2004). The N- and C-terminus as well as the hook region connecting the SH3 and GK domains are highly variable among the four genes and subject to alternative splicing (Buraei and Yang, 2010). In heterologous cells co-expression of a β subunit is crucial for functional membrane expression of α_1_ subunits (Buraei and Yang, 2010). Initially it has been proposed that β promotes membrane expression by masking an ER retention signal in the α_1_ subunit (Bichet et al., 2000). More recent evidence supports a more complex model according to which β binding to the AID shifts the balance between multiple ER export and retention signals (Fang and Colecraft, 2011). In addition to increasing the current amplitude by promoting membrane incorporation of functional channels, co-expression of β subunits left-shifts the voltage-dependence of current activation (Buraei and Yang, 2010). β_2a_ and β_2e_ differ from the other β subunits in that they contain an N-terminal palmitoylation or a positively charged sequence, respectively, that target them to the plasma membrane and upon co-expression with α_1_ subunits dramatically slow down the rate of current inactivation (Olcese et al., 1994; Chien et al., 1998; Miranda-Laferte et al., 2014). In native cells β subunits may play additional roles in subcellular targeting and in the specific incorporation of the channel in macromolecular signaling complexes (see below).

The original purifications of VGCCs from skeletal muscle revealed yet another auxiliary subunit, the transmembrane γ_1_ protein. The γ subunit is an integral membrane protein with four transmembrane helices (Chen et al., 2007). In skeletal muscle the γ_1_ subunit functions to limit both, the calcium current and depolarization-induced SR calcium release by stabilizing the inactivated state of Ca_V_1.1 (Andronache et al., 2007). It appears therefore that inclusion of a γ subunit in the Ca_V_ complex is specific to skeletal muscle and γ subunits are not necessarily required for functional expression of Ca_V_ channels in heterologous cells. In contrast, γ_1_ belongs to a family of eight genes predominantly expressed in the brain where they are known as transmembrane AMPA receptor regulatory proteins (TARPs) (Catterall, 2011). They associate with the glutamate receptor and play an important role in targeting and anchoring these ionotropic receptors in the postsynaptic membrane of neurons as well as in glia cells (Chen et al., 2007). A recent study demonstrated that different γ subunits differentially modulate the inactivation properties of the cardiac Ca_V_1.2 channel in HEK293 cells (Yang et al., 2011). However, apart from the Ca_V_1.1/γ_1_ interaction in skeletal muscle, it is not known whether γ and α_1_subunit isoforms functionally interact with each other in native tissues.

### What defines an auxiliary calcium channel subunit?

The complement of canonical VGCC subunits was originally defined by their co-purification from muscle tissue. This definition has served us well in that it described a minimum set of proteins that are part of many Ca_V_ channels and more or less important for their normal functions. However, since then many other calcium channel interacting and modulating proteins have been identified, and conversely the known roles of the classical channel subunits are no longer limited to Ca_V_ channel functions. These developments raise the question as to which properties actually define a protein as a calcium channel subunit. Is it its co-purification with an α_1_ subunit or its functional interaction with the channel, the exclusiveness of its association with Ca_V_ channels or the absolute requirement for its function, or a combination of any or all of these properties? No matter which of these properties one would consider, no definition would include all classical auxiliary subunits or necessarily exclude other VGCC-interacting proteins.

For example today we know that T-type (Ca_V_3) channels do not contain any of the classical auxiliary subunits and that inclusion of the γ subunit may be specific to Ca_V_1.1 channels in skeletal muscle (Zhang et al., 2013). Thus, the classical VGCC subunits are not universally associated with all members of the Ca_V_ family. The classical VGCC subunits also fall short of being exclusive to Ca_V_ channels. Based on the number of γ genes expressed in the brain versus muscle and on their importance as AMPAr regulatory proteins, γ proteins primarily have to be considered as being AMPAr subunits, with one notable exception, γ_1_, which associates with a calcium channel (Chen et al., 2007). But also for α_2_δ and β subunits Ca_V_ channel-independent functions have been reported, indicating that their functional importance and molecular interactions may not be limited to Ca_V_ channels (Eroglu et al., 2009; Subramanyam et al., 2009).

Also the importance of the classical auxiliary subunits for specific Ca_V_ channel-dependent functions differs greatly. Although β subunits are important for normal surface expression and current properties of Ca_V_ channels in most expression systems, α_2_δ subunits seem to be less important and γ subunits even dispensable for normal muscle function (Obermair et al., 2008; Catterall, 2011). On the other hand the ubiquitous calcium sensing protein calmodulin is associated with Ca_V_1 and Ca_V_2 channels and plays an important role in feedback regulation (i.e., calcium-dependent inactivation and facilitation) as well as in the subcellular localization of Ca_V_ channels (Wang et al., 2007; Minor and Findeisen, 2010). Yet, it has no rank among the canonical auxiliary calcium channel subunits. Calmodulin belongs to a group of VGCC associated proteins, which are constitutively associated with most, if not all, Ca_V_1 and Ca_V_2 channels and modulate channel expression as well as their current properties. Therefore we will refer to these proteins as non-canonical calcium channel subunits (see Section non-canonical auxiliary calcium channel subunits).

Apparently the importance of an associated protein for Ca_V_ channel function is neither necessary nor sufficient to define it as auxiliary calcium channel subunit. Rather the current definition of α_2_δ, β, and γ as auxiliary calcium channel subunits is mostly based on historical reasons. However, 25 years of calcium channel research have led to a much more complex picture of auxiliary calcium channel proteins. Therefore it is necessary to re-think the physiological role of auxiliary calcium channel subunits and perhaps question some of the cherished traditional concepts.

## Auxiliary Subunits Increase the Molecular Diversity of Calcium Channels

Mammals have four genes each encoding for α_2_δ and β subunits (Fig. 1C), both with several known splice variants and probably many more to be identified (Buraei and Yang, 2010; Dolphin, 2013). (Because of the limited importance of the γ_1_ as calcium channel subunit we will from here on focus our discussion on α_2_δ and β subunits.) Counting only the number of α_2_δ and β genes together with the seven Ca_V_1 and Ca_V_2 α_1_ subunit genes, there are 112 possible subunit combinations. Further considering the multiple splice variants of each gene we would likely arrive at a number of over thousand possible molecularly distinct calcium channel combinations. Do we need such a great molecular diversity and if so, for what purpose?

The calculation above is based on the assumption that every subunit variant can associate with every α_1_ subunit. Therefore, we first need to question whether such unlimited subunit combinations are at all possible. A host of co-expression studies in heterologous cells support the concept of a great promiscuity in calcium channel subunit interactions (Obermair and Flucher, 2013). In fact, the authors of this review are not aware of a single study demonstrating that a given α_2_δ or β subunit cannot functionally interact with any specific Ca_V_1 or Ca_V_2 α_1_ subunit, when heterologously co-expressed. There is also evidence that a similar heterogeneity in channel interactions occurs in differentiated and native cell systems (Obermair et al., 2010; Campiglio et al., 2013). In those cases where isolated channel combinations serve specific cellular functions this typically is linked to the co-expression of a unique set of channel isoforms (Obermair and Flucher, 2013; Geisler et al., 2014).

The overwhelming functional evidence for highly promiscuous subunit interactions is supported by the available studies measuring binding affinities between α_1_ and β subunits, or β subunits and AID peptides (Van Petegem et al., 2008). AID binds different β isoforms with affinities in the low nM range and any isoform-specific differences in the strength of binding are rather small (Buraei and Yang, 2010). Together these studies do not suggest that isoform-specific subunit combinations form spontaneously in vitro or when expressed in heterologous cells. Thus, we need to consider the possibility of an extensive molecular diversity of VGCC subunit combinations.

However, cell- and tissue-specific expression patterns or differential developmental expression of the calcium channel subunit isoforms can limit the extent of possible subunit combinations in specific cell types (Obermair and Flucher, 2013; Geisler et al., 2014). For example skeletal muscle exclusively expresses Ca_V_1.1, α_2_δ-1 and β_1a_, whereas heart muscle expresses Ca_V_1.2, α_2_δ-1 and β_2b_. The hair cells of the inner ear express Ca_V_1.3, α_2_δ-3 and β_2_, whereas retinal photoreceptor cells express Ca_V_1.4 and α_2_δ-4 (two isoforms that seem to be unique to this tissue) and β_2_ (Lee et al., 2015). Since these cells express only a single isoform of each subunit, null-mutants of the respective gene result in the corresponding disease phenotypes (Gregg et al., 1996; Neef et al., 2009; Stockner and Koschak, 2013). Some neuron types show a predominance of specific channel subunits. For example cerebellar granule cells express high levels of Ca_V_2.1 together with β_4_ and α_2_δ-2 (Schlick et al., 2010). Although in such neurons other isoforms may be expressed at lower levels, these cannot fully compensate the loss of any one of the dominant isoforms. Accordingly, null-mutants of any of these dominant calcium channel subunit genes—in tottering (Ca_V_2.1), ducky (α_2_δ-2), and lethargic (β_4_) mice—result in very similar neurological defects characterized by ataxia and cerebellar epilepsy (Felix, 2002). On the other hand, the majority of neurons in the central nervous system expresses multiple isoforms at comparable levels, allowing a great molecular diversity of calcium channel combinations and loss of a single isoform readily can be compensated by the other isoforms (Schlick et al., 2010; Obermair and Flucher, 2013; Geisler et al., 2014).

Similarly the expression of some calcium channel subunit isoforms is limited to specific stages of development. Developing and differentiated cells have different requirements for calcium signaling that are reflected in the expression of distinct Ca_V_ channel isoforms. Recently we identified a splice variant of Ca_V_1.1 which is the dominant isoform in embryonic muscle but is almost exclusively replaced by expression of a functionally very distinct Ca_V_1.1 splice variant in mature skeletal muscle (Tuluc et al., 2009). Another example is provided by the expression of β_2c_, β_2d_, and β_2e_ in embryonic cardiac muscle, whereas in adult heart expression of these isoforms declines and β_2b_ becomes the predominant β subunit (Buraei and Yang, 2010).

Together these examples demonstrate that differential expression of calcium channel isoforms and splice variants gives rise to both spatially and temporally distinct calcium channel complexes. These mechanisms dramatically decrease the number of possible subunit combinations within a given cell. Nevertheless, in many cell types the simultaneous expression of multiple α_2_δ and β isoforms and their promiscuous association with several expressed α_1_ subunits still allows for a considerable combinatorial complexity and molecular diversity of voltage-gated calcium channels in many excitable cells.

### What is the functional importance of the molecular diversity of auxiliary subunits?

Usually molecular diversity is equated with functional diversity. With regard to calcium channels the studies of subunit interactions are strongly grounded on the belief that association of a given α_1_ subunit with alternative β or α_2_δ subunits endows the channel with different current properties. This concept is supported by the distinct voltage sensitivity of activation and the slow inactivation kinetics found in channels co-expressed with the membrane-associated β_2a_ or β_2e_ isoforms compared to those of other isoforms (Olcese et al., 1994; Miranda-Laferte et al., 2014). In contrast to β_2a_ and β_2e_ all other examined β isoforms, or mutated β_2a_ lacking the N-terminal palmitoylation site, show very similar effects on voltage-dependence and current density (Buraei and Yang, 2010). Likewise, also different α_2_δ subunit isoforms appear to exert very similar effects upon co-expression with specific α_1_ subunits. Apparently, the distinct effects on Ca_V_ current properties of the membrane-associated β_2a_ and β_2e_ are the exception among the auxiliary subunits, not the rule. Therefore, the concept that the large molecular diversity among auxiliary subunits exists in order to tune current properties to the specific requirement may need to be abandoned in light of the paucity of evidence obtained in 25 years of co-expression studies.

If two molecularly distinct subunit isoforms or splice variants endow a channel with similar functional properties, they will create functional redundancy rather than functional diversity. Functional redundancy among different β and α_2_δ isoforms in vivo is supported by evidence from several mutant animal models. Whereas null-mutants of some β and α_2_δ subunit genes do show disease phenotypes, these are for the most part associated with calcium channel functions in cells or tissues that express only a single isoform (Obermair and Flucher, 2013; Geisler et al., 2014). However, in the brain, where the majority of cells express multiple α_2_δ and β isoforms these knockout animal models cause little or no defects (Buraei and Yang, 2010; Dolphin, 2013). Apparently, other isoforms are functional redundant and thus can compensate the loss of the mutated subunit isoform. As the studies in the knockout mouse models show, functional redundancy increases the safety factor for cellular functions and thus may by itself represent a benefit of molecular diversity. Whereas this may explain why some cells express more than one auxiliary subunit gene, it appears to be a poor explanation for the large molecular diversity among α_2_δ and β genes and certainly does not explain the need for multiple splice variants, which often would be equally affected by genetic defects.

One alternative explanation might be that distinct auxiliary subunits can differentially interact with proteins involved in targeting of VGCCs or in the formation of signaling complexes. In polarized epithelial MDCK cells recombinant Ca_V_2.1 is targeted to the apical membrane when co-expressed with β_1b_ or β_4_, while it is trafficked to the basolateral membrane when co-expressed with β_2a_ (Brice and Dolphin, 1999). This study suggested for the first time that different β isoforms could target calcium channels to structurally and functionally distinct membrane domains of polarized cells. A study from our laboratory reported differential localization of various β isoforms in cultured hippocampal neurons (Obermair et al., 2010). In fact, while all examined β isoforms were incorporated in the presynaptic terminals, β_1_ isoforms were transported poorly to the distal axons compared to the other β isoforms. This indicates that specific axonal targeting mechanisms may in part specify the subunit combination of presynaptic calcium channels. This notion is further substantiated by a recent study demonstrating differential somato-dendritic and axonal targeting properties of three β_4_ splice variants in differentiated neurons (Etemad et al., 2014b). Differential axonal targeting properties may also enrich specific α_2_δ subunits in the presynaptic compartment. Unpublished data from the laboratory of G. Obermair also indicate differential axonal targeting properties in α_2_δ subunit isoforms (personal communication). Together these studies provide evidence that individual channel subunits are specifically targeted to the presynaptic compartment, where they may assemble into specific channel complexes based on their relative availability.

Theoretically, specific β subunit targeting mechanisms might function to target entire channel complexes into specific subcellular compartments. However, axonal targeting of preassembled channel complexes again would require their specific assembly in the soma, where a multitude of channel subunits are present. Given the promiscuous association this is unlikely to create the highly specific composition of presynaptic channels. Moreover, to date there is no compelling evidence for the existence of an auxiliary subunit guided targeting mechanism. In contrast, β subunits require the α_1_ subunits for their own subcellular targeting, not vice versa. For example, without an α_1_ subunit β subunits fail to incorporate in the triad junctions of skeletal myotubes (Neuhuber et al., 1998; Campiglio et al., 2013). When an α_1_ subunit is expressed and targeted into the junctions, any co-expressed β subunit associates with it (Subramanyam et al., 2009; Campiglio et al., 2013; Etemad et al., 2014). Similarly the subcellular localization of β subunit in neurons chiefly depends on the interaction with specific α_1_ subunits (Obermair et al., 2010). Thus, existing evidence suggests that specific targeting of auxiliary subunits to a subcellular compartment may contribute to the generation of specific subunit composition of the channel complexes in the presynaptic membrane. Obviously, such a mechanism would be limited to neurons or a few other cell types where a similar spatial segregation of channel subunits by specific targeting mechanisms is possible.

An alternative but plausible explanation for a high subunit complexity is that different auxiliary subunits might specifically interact with distinct up- or down-stream signaling proteins. Thereby channel complexes containing distinct auxiliary subunits could be differentially modulated or initiate differential cellular responses. Below, we describe several such VGCC signaling complexes and review the experimental evidence in support of the notion that auxiliary calcium channel subunits function as important coordinators of these signaling complexes, in part in an isoform-specific manner.

## VGCCs Function in the Context of Calcium Channel Signaling Complexes

Calcium-mediated signaling processes achieve their remarkable speed and specificity by a tight spatio-temporal regulation of the calcium signal. For less than a millisecond calcium concentration near the cytoplasmic mouth of VGCC can rise by three orders of magnitude (from 100 μM to 100 mM) before it dissipates due to diffusion, buffering, and active removal processes. Consequently, to become activated, low affinity calcium sensing proteins need to be located within the close vicinity of the channel. Therefore many VGCCs are colocalized with calcium sensors, regulatory proteins, and effector proteins in macromolecular signaling complexes encompassing calcium micro- and nanodomains. In this context VGCCs not only function as voltage sensors and the calcium source, but also serve as signaling platforms for up- and down-stream pathways (De Waard et al., 1996; Tsien and Barret, 2005). The large cytoplasmic domains of the α_1_ subunits with multiple protein–protein interaction domains provide many binding sites for modulatory and effector proteins (Dai et al., 2009). Furthermore, auxiliary subunits greatly increase the potential of VGCCs to form transmembrane signaling complexes. The intracellular β subunit belongs to the MAGUK family of scaffold proteins containing an SH3 and a catalytically inactive GK domain, which has evolved into a highly specific protein interaction domain (Buraei and Yang, 2010). In contrast, the relatively short extracellular domains of the pore-forming subunit provide few potential interaction sites. However, the potential for extracellular interactions is substantially increased by the α_2_δ subunit, which is completely extracellular. α_2_δ is highly glycosylated and contains a von Willebrand factor type A (VWA) cell–cell adhesion domain that is typically found in extracellular matrix proteins and integrin receptors (Geisler et al., 2014). Consequently, the α_2_δ-α_1_-β channel complex comprises a platform for transmembrane signaling in which the α_1_ subunit primarily determines the specific gating and current properties and the different α_2_δ while β subunit isoforms determine the subcellular localization and the molecular context for the calcium signal.

In the following sections we will describe macromolecular signaling complexes formed by calcium channels, and present evidence indicating that the auxiliary subunits serve as scaffold proteins in these VGCC complexes. In some cases specific auxiliary subunit isoforms determine whether particular proteins associate with the signaling complex or whether a channel complex is sensitive to a particular mode of modulation or not (summarized in Fig. 2 and Table1). These findings suggest the importance of the molecular subunit diversity in the formation of various functional calcium channel signaling complexes.

**Table 1:** β-Dependent VGCC interactions

Protein	β isoform in the VGCC	Interaction partner	Function	Reference
RIM1	any β isoform	Ca_V_2	Targeting and docking of vesicles in proximity to VGCCs	Coppola et al. (2001)
RGK	any β isoform	Ca_V_1, Ca_V_2	Current inhibition	Yang and Colecraft, (2013)
PKC	any β isoform	Ca_V_2.2, Ca_V_2.3	Current increase and relief of G_βγ_ inhibition	Stea et al., (1995)
RyR1	β_1a_	Ca_V_1.1	Essential for skeletal muscle ECC	Gregg et al. (1996); Schredelseker et al. (2005)
PKA	β_1b_>β_3_, β_4_>β_2a_	Ca_V_1.2	Current increase during β-adrenergic	Miriyala et al. (2008)
	β_2a_>β_3_	Ca_V_1.3	Stimulation	Liang and Tavalin (2007)
Ahnak1	β_2_	Ca_V_1.2	PKA upregulation in heart	Pankonien et al. (2012)
Synaptotagmin I	β_3_, β_4a_, not β_4b_	Ca_V_2	Targeting and docking of vesicles in proximity to VGCCs	Vendel et al. (2006)
G_βγ_	β_3_>β_4_>β_1b_>β_2a_	Ca_V_2	Current inhibition	Feng et al., (2001); Buraei and Yang (2010)
CaMKII	β_1b_, β_2a_, not β_3_, β_4_	Ca_V_1, Ca_V_2	Current increase and CDF	Buraei and Yang (2010)
erbin	β_1b_, not β_4_	Ca_V_1.3	VDF	Calin-Jageman et al. (2007)
AA	β_2a_	Ca_V_1.3	Current inhibition	Roberts-Crowley and Rittenhouse (2009)

### The excitation-contraction (EC) coupling apparatus

The best described VGCC-mediated mechanism is EC coupling, the process in which an electric signal, the action potential, is converted into muscle contraction. This fundamental process in muscle physiology is mediated by Ca_V_1.1 and Ca_V_1.2 VGCCs, also called dihydropyridine receptors (DHPR), which in response to membrane depolarization activate channel opening and the opening of calcium release channel (ryanodine receptors or RyR) in the sarcoplasmic reticulum (SR). EC coupling takes place in junctions between the SR and the plasma membrane or its invaginations the T-tubules, where Ca_V_1 channels and the RyR are located in direct apposition. Different subunit compositions of Ca_V_1 and the RyR in skeletal and cardiac muscle determine distinct molecular signaling mechanisms (Franzini-Armstrong et al., 1998).

In skeletal muscle the DHPR is composed of Ca_V_1.1, α_2_δ-1, and β_1a_, which physically interact with the type 1 RyR. Upon depolarization the Ca_V_1.1 undergoes a conformational change which leads to the opening of the RyR1. The physical interaction of Ca_V_1.1 and RyR1 is also reflected by the organization of Ca_V_1.1 in ordered tetrads arrays (groups of four) opposite to one RyR, which can be visualized in freeze–fracture electron microscopy. Influx of calcium through the Ca_V_1.1 channel is not necessary for functional EC coupling and in many fish (e.g., zebrafish) Ca_V_1.1 is non-conducting and functions merely as voltage sensor (Schredelseker et al., 2010). The essential features for skeletal muscle EC coupling and for tetrad formation (i.e., the correct incorporation into the functional signaling complex) are the II-III intracellular loop of Ca_V_1.1 and the SH3 domain and C-terminus of β_1a_. In fact, EC coupling fails in null mutants of either Ca_V_1.1 or β_1_. A chimera of Ca_V_1.2 containing the II-III loop of Ca_V_1.1 can reconstitute EC coupling and tetrad formation in Ca_V_1.1-null myotubes (Grabner et al., 1999; Wilkens et al., 2001; Kugler et al., 2004). Similarly, a chimera of β_3_ containing the SH3 domain and the C-terminal region of β_1a_ can fully restore EC coupling in relaxed β_1_-null myotubes (Dayal et al., 2013). Importantly, the specific β subunit isoform is critical for tetrad formation and thus for the direct Ca_V_1.1-RyR1 coupling mechanism in skeletal muscle (Schredelseker et al., 2005). In contrast, the α_2_δ-1 subunit seems to be dispensable for EC coupling (Obermair et al., 2005).

Recent reports revealed a hitherto unnoticed essential component of the EC coupling machine. STAC3 is a cytoplasmic Ca_V_1.1 binding protein containing a cysteine rich region and two SH3 domains. It is necessary for membrane expression of Ca_V_1.1 in heterologous cells and its deletion in skeletal muscle impairs EC coupling in mouse and zebrafish (Horstick et al., 2013; Nelson et al., 2013; Polster et al., 2015). Both, the β_1a_ subunit and STAC3 associate with the Ca_V_1.1 α_1S_ subunit, whereas their possible interactions with the RyR1 depend on the presence of Ca_V_1.1 (Campiglio et al., 2013; Polster et al., 2015). Thus, it appears that β_1a_ and STAC3 are important for the correct assembly of the skeletal muscle EC coupling apparatus. Whether they also participate in the signal transduction process remains controversial.

Whereas skeletal muscle EC coupling is independent of calcium influx, the β-adrenergic receptor mediated increase in the force of the contraction depends on extracellular calcium. At rest the C-terminus of Ca_V_1.1 is proteolytically cleaved but remains associated with the channel, forming an autoinhibitory complex with reduced calcium currents. Upon activation of the β-adrenergic/PKA signaling pathway both the C-terminus of Ca_V_1.1 and β_1a_ are phosphorylated (Catterall, 2010). PKA dependent phosphorylation of the Ca_V_1.1 C-terminus reverses its autoinhibitory function and the calcium current and EC coupling are augmented. This signaling mechanism critically depends on the anchoring of PKA to the C-terminus of Ca_V_1.1 by the adaptor protein AKAP15 (Catterall, 2010).

In cardiac muscle EC coupling is carried out by a channel complex consisting of Ca_V_1.2, α_2_δ-1, mainly β_2b_ (but also β_1d_, β_2a-d_, and β_3_) and the RyR2. Ca_V_1.2 and RyR2 are also localized in close proximity in the junctions between the sarcoplasmic reticulum and the plasma membrane, but unlike in skeletal muscle, they are not directly coupled. As a consequence the DHPRs do not form tetrads and cardiac EC coupling requires calcium influx through Ca_V_1.2 (Franzini-Armstrong et al., 1998) Thus, Ca_V_1.2 calcium triggers the opening of RyR2 in a process called calcium-induced calcium release (CICR). Importantly, CICR in cardiac muscle also necessitates the colocalization of the Ca_V_1.2 channel in the close proximity of the RyR2. Heterologously expressed Ca_V_2.1 channels, which conduct even larger currents but are not targeted into triad junctions, fail to induce CICR in dysgenic myotubes (Tanabe et al., 1990). Therefore, even without a direct physical link between Ca_V_1.2 and RyR2, these channels form a functional signaling complex.

Like in skeletal muscle this calcium channel signaling complex is modulated by β-adrenergic stimulation in the fight or flight response. Again the mechanism involves the relief of an autoinhibitory protein–protein interaction within the C-terminus of Ca_V_1.2, as well as the association of PKA by AKAP15. In vitro PKA also phosphorylates three sites of β_2a_ (Gerhardstein et al., 1999). However, these phosphorylation sites were shown to be not essential in the PKA upregulation of L-type current in cardiac myocytes (Miriyala et al., 2008). Nevertheless, the β subunits play an important role in the β-adrenergic facilitation of Ca_V_1.2 currents. The extent of PKA modulation is highest with β_1b_, followed by β_3_ and β_4_, while β_2a_ shows the weakest modulation. As PKA modulation of Ca_V_1.2 signaling also can occur also in brain the differential effects of different β subunits may be involved in the tissue-specific tuning of this modulatory process (Miriyala et al., 2008).

In the heart the large membrane scaffold protein Ahnak1 has been suggested to play a role in the β-adrenergic modulation of Ca_V_1.2. Ahnak1 contains docking sites for several cytoskeleton proteins and for auxiliary β subunits. It has been suggested that Ahnak1 binds to the GK domain of β_2_, acting as a repressor of the β_2_ functionality during basal conditions (Pankonien et al., 2012). Upon PKA activation β_2a_ is phosphorylated at Ser296, which reduces its binding to Ahnak1 allowing full augmentation of calcium influx (Pankonien et al., 2012). Together these examples indicate that in cardiac muscle, in which calcium influx through Ca_V_1.2 controls the force of contraction, auxiliary β subunits are critically involved in the current modulation by functionally and structurally interacting with various signaling and scaffold proteins.

### The presynaptic calcium channel signaling complex

One of the most studied mechanisms controlled by VGCCs is regulated secretion and specifically presynaptic neurotransmitter release. Presynaptic Ca_V_2 channels conduct P/Q- and N-type calcium currents, which trigger the fusion of synaptic vesicles with the plasma membrane. The efficiency of synaptic release critically depends on the amount of calcium entering the nerve terminal and on the proximity of the calcium channels to the fusion apparatus. The synaptic vesicle membrane contains the v-SNARE protein VAMP/synaptobrevin, which interacts with two t-SNARE proteins of the plasma membrane, SNAP-25 and syntaxin. The calcium sensor protein synaptotagmin I, a transmembrane protein that binds syntaxin and the SNARE complex, triggers vesicle fusion upon calcium binding (Catterall, 2011). The colocalization of the VGCCs and the fusion apparatus within a calcium nanodomain allows transmitter release within 200 μs of the action potential (Sudhof, 2013).

Presynaptic VGCCs directly interact with the synaptic vesicle fusion apparatus through a specific interaction site called synprint, located in the II-III loop of the α_1_ subunit. Synprint has been suggested to interact with syntaxin, SNAP25 and synaptotagmin I in a calcium- and phosphorylation-dependent manner (Sheng et al., 1998), and it has also been shown to be important for synaptic targeting of calcium channels (Szabo et al., 2006). Synaptotagmin I also binds to specific β isoforms (β_3_ and β_4a_ but not β_4b_) (Vendel et al., 2006). Furthermore the Rab-interacting molecule 1 (RIM1), a presynaptic scaffold protein which binds the synaptic vesicle protein Rab3, also binds to auxiliary β subunits, and thus may be involved in the targeting and docking of vesicles in proximity of the VGCCs. RIM1 binding to the auxiliary β subunits not only has a scaffolding function, but also modulates the current properties of the channel. It slows down voltage-dependent inactivation and shifts its voltage-dependence to hyperpolarizing potentials (Coppola et al., 2001). These findings indicate a dual function of auxiliary β subunits in anchoring synaptic vesicles to presynaptic VGCCs and in the modulation of the calcium current that triggers vesicle fusion.

It has also been suggested that the auxiliary subunit α_2_δ plays a role in targeting VGCC to the presynaptic boutons (G. Obermair, personal communication), presumably through interactions with extracellular active-zone-specific proteins (Geisler et al., 2014). In the nerve terminal α_2_δ may be involved in coupling presynaptic Ca_V_ to the synaptic release machinery (Hoppa et al., 2012). Furthermore, α_2_δ subunits have been suggested to play a role in synaptogenesis. The α_2_δ-1 subunit has been identified as a receptor of thrombospondin, an astrocyte-secreted protein that promotes synaptogenesis (Eroglu et al., 2009). In that study overexpression or knockdown of α_2_δ-1, respectively, promoted or inhibited excitatory synapse formation in cultured retinal ganglion cells.

Auxiliary β subunits may play a crucial role not only in the targeting of presynaptic VGCCs in the proximity of the synaptic release machine, but also in the tuning of the VGCC properties. Because of their crucial role in synaptic release, presynaptic calcium channels are subject to regulation by several modulatory pathways. For example Ca_V_2 channels undergo negative-feedback inhibition through G-protein coupled receptor pathways, which represent mechanisms of presynaptic inhibition and short-term synaptic plasticity (Buraei and Yang, 2010). Nerve terminals contain G protein-coupled receptors that are activated by spillover of neurotransmitters and provide negative feedback by inhibiting the presynaptic calcium channels; thus, reducing neurotransmitter release. Specifically the G_βγ_ dimer has been demonstrated to bind directly to Ca_V_2 channels at multiple sites, including two in the I-II loop, one up-stream and one down-stream of the I-II loop. Binding of G_βγ_ to Ca_V_2 channels results in a shift in the voltage-dependence of activation to more depolarized potentials and a slowing of activation kinetics. The auxiliary β subunit appears to be essential for this inhibition (Buraei and Yang, 2010). When the β subunit binds to the AID, the I-II loop adopts a rigid α-helical structure and this structural property is crucial for the G_βγ_ inhibition. Furthermore it was demonstrated that distinct β isoforms have different effects on the extent of the G_βγ_ inhibition, with β_3_ being the most effective (Feng et al., 2001). The presence of an auxiliary β subunit is also essential also for PKC modulation of specific Ca_V_ subunits (Stea et al., 1995). PKC activity can relieve G_βγ_ inhibition, by increasing the activity of R-type and N-type channels, which potentiate synaptic release. Upon activation PKC phosphorylates Ca_V_2 channels on the I-II loop, which releases the G_βγ_ dimer and abolishes its inhibition on the channel activity in a β-dependent manner (Dai et al., 2009).

Together these examples highlight the importance of the auxiliary calcium channel subunit in presynaptic function. They are involved in the molecular interactions regulating the tightness of coupling between excitation and synaptic release, as well as in orchestrating up-stream signaling pathways, which dynamically regulate the release probability by modulating the gating properties of the presynaptic channels. Emerging evidence indicates isoform-specificity of these functions. Interestingly, a proteomics approach identified around 200 proteins as potential interaction partners of neuronal Ca_V_2 channel complexes, but only less than 10% of these were known presynaptic signaling proteins (Muller et al., 2010). Apparently the complexity of the presynaptic calcium channel signaling machinary vastly exceeds our current models.

### The postsynaptic calcium signaling complex

The postsynaptic Ca_V_ channels (predominantly Ca_V_1.2 and Ca_V_1.3) are localized in the soma of neurons as well as in dendritic shafts and spines (Obermair et al., 2004). There they contribute to the neuronal excitability and they activate the excitation-transcription coupling pathway. The postsynaptic channels form macromolecular signaling complexes and require interactions with specific proteins for their own proper function (Calin-Jageman and Lee, 2008; Striessnig et al., 2014).

The C-terminus of Ca_V_α_1_ subunits is a major modulatory domain that contains multiple binding sites for regulatory proteins and for scaffold proteins. As mentioned in section 3.1, the distal C-terminus of Ca_V_1.2 as well as that of Ca_V_1.3 can be cleaved, but remains non-covalently associated to the proximal C-terminus. This intramolecular interaction exerts an autoinhibitory effect and reduces the channel activity by more than 10-fold (Hulme et al., 2006; Striessnig et al., 2014). PKA is anchored to the C-terminus of the channels through AKAP15, and upon activation phosphorylates the proximal C-terminus and disinhibits Ca_V_1.2 and Ca_V_1.3 currents (Dai et al., 2009; Catterall, 2010). PDZ-binding motifs in the very C-terminus of Ca_V_1 channels seem to be important for coordinating calcium channel signaling complexes in the postsynaptic compartment of neurons (Stanika et al., 2015). For example, the PDZ domain-containing protein erbin mediates voltage-dependent facilitation (VDF) of Ca_V_1.3 channels, by relieving the autoinhibitory action of the C-terminus (Calin-Jageman et al., 2007). Importantly, erbin’s effect on the Ca_V_1.3 current properties critically depends on the auxiliary β subunit isoform in the complex. It augments VDF of Ca_V_1.3 channels containing β_1b_, but not of those containing β_4_ (Calin-Jageman et al., 2007). Densin, another PDZ domain containing protein, has been reported to anchor the calcium/calmodulin protein kinase II (CaMKII) to Ca_V_1.3 and thereby promote calcium-dependent facilitation (CDF) during high-frequency stimulation (Jenkins et al., 2010). CaMKII activity also regulates Ca_V_1.2 and Ca_V_2.1 channels, slowing down inactivation and positively shifting their voltage-dependence (Dzhura et al., 2000; Jiang et al., 2008). Interestingly, CaMKII binds in vitro to β_1_ but not to β_3_ and β_4_ (Grueter et al., 2008), and it co-immunoprecipitates with forebrain Ca_V_1 channel complexes containing β_1b_ or β_2a_, but not with those containing β_4_ (Abiria and Colbran, 2010).

A different signaling mechanism regulating VGCC activity are the membrane lipids and their metabolic products, like arachidonic acid (AA). AA inhibits the calcium current of all VGCC, including the Ca_V_3 channels which do not contain auxiliary subunits. Therefore one could speculate that the auxiliary subunits play no role in the AA inhibition of calcium currents. However, when a Ca_V_1.3 channel complex contains β_2a_, AA exerts little inhibition. Interestingly this dampening of the AA action critically depends on the palmitoylation of β_2a_ (Roberts-Crowley and Rittenhouse, 2009). This suggests that even in regulatory pathways that act directly on the α_1_ subunit, auxiliary subunits can modify the extent of the modulation of the channel properties. These are striking examples demonstrating how different β isoforms are involved in mediating specific up-stream signaling pathways that modulate neuronal calcium channel function; but auxiliary subunits are similarly involved in down-stream signaling pathways.

### The excitation-transcription coupling pathway

VGCCs are important regulators of activity-dependent regulation of gene expression in excitable cells. Since Ca_V_1 channels are preferentially localized in the soma and proximal dendrites of neurons, they have a privileged role in activity-dependent signaling to the nucleus, as opposed to the predominantly presynaptic Ca_V_2 channels and the NMDA receptor (Bading et al., 1993; Dolmetsch et al., 2001; Di Biase et al., 2008). VGCC regulate transcription via activation of classical signal transduction cascades and by nuclear targeting of channel fragments or subunits.

Calmodulin, the ubiquitous and yet neglected calcium channel subunit, plays a central role in gene regulation in neurons (Bito et al., 1996; Dolmetsch et al., 2001). Calcium-calmodulin activation of CREB has been implicated in a wide array of neuronal functions, such as survival, neuronal morphology and synaptic plasticity (Lonze and Ginty, 2002). Calcium entering the cell through VGCC binds calmodulin and activates CaMKI, CaMKII and CaMKIV. Considering the differential effects of β isoforms on CaMKII function (see previous paragraph), it will be interesting to investigate β specificity in CaMKII mediated transcriptional signaling as well. Ca_V_-dependent calcium influx also activates the calcium/calmodulin dependent protein phosphatase calcineurin (CN). CN is anchored to the C-terminus of Ca_V_1 channels by the scaffolds protein AKAP79/150. In cultured hippocampal neurons, desphosphorylation of the transcription factor NFATc4 by CN results in its migration of the nucleus, where it regulates transcription (Oliveria et al., 2007).

In addition to these classical calcium-dependent signaling pathways, VGCC can directly regulate transcription by the nuclear targeting of channel fragments or β subunits. The C-terminus of Ca_V_1.2 was detected in the nuclei of neurons where it appears to regulate transcription. In the nucleus the Ca_V_1.2 C-terminus binds to the transcriptional regulator p54 (nrb/NonO) and to the enhancer region of the connexin 3.1 gene (Gomez-Ospina et al., 2006). In cardiac myocytes, the distal C-terminus of Ca_V_1.2 acts as a repressor of the Ca_V_1.2 promoter, suggesting that Ca_V_1.2 autoregulates its own expression (Schroder et al., 2009). Furthermore, a C-terminal fragment of Ca_V_2.1 has been found to translocate to the nuclei of cerebellar nuclei (Kordasiewicz et al., 2006). This nuclear targeted C-terminus has been implicated in the cytotoxic effect of spinocerebellar ataxia type 6 Ca_V_2.1 mutations which carry an expanded polyglutamine stretch in their C-terminus.

A similar channel-independent function in transcriptional regulation has been reported for auxiliary β subunits. The first β isoform found to translocate to the nucleus and regulate gene transcription was β_4c_, a truncated β_4a_ variant isolated from chicken cochlea. In the nucleus, β_4c_ interacts with the chromobox protein 2/heterochromatin protein 1 (CHCB2/HP1γ), decreasing its gene silencing activity and thus promoting gene expression (Hibino et al., 2003). The corresponding β_4c_ isoform was subsequently isolated from human brain, where it is prominently expressed in vestibular neurons (Xu et al., 2011). Immunohistochemistry analysis of cerebellar granule and Purkinje cells of mice revealed β_4_ in the nucleus (Subramanyam et al., 2009). Of the β_4_ splice variants particularly β_4b_ showed nuclear targeting when expressed in neurons, skeletal muscle or in HEK cells (Subramanyam et al., 2009; Etemad et al., 2014b). A chimeric approach revealed that an N-terminal double arginine motif confers the nuclear targeting property to β_4b_ (Subramanyam et al., 2009). However, the mechanism of nuclear localization of β_4b_ remains controversial: while our group demonstrated in multiple studies that the nuclear localization of β_4b_ is negatively regulated by spontaneous electrical activity and calcium influx (Subramanyam et al., 2009; Etemad et al., 2014; Etemad et al., 2014b), another group suggested that the accumulation of β_4b_ in the nucleus is activated by membrane depolarization (Tadmouri et al., 2012). These authors also proposed a mechanism by which in response to VGCC activation β_4b_ recruits the B56δ/PP2A complex and translocates to the nucleus. There it promotes histon modification and interacts with the transcription factor TRα, inhibiting the transcription of the tyrosine hydroxylase (TH) gene transcription. An Affymetric GeneChip analysis on mRNA extracts from β_4_-null cerebellar granule cells reconstituted with three different β_4_ splice isoforms revealed that their differential ability for nuclear targeting correlates with their potential to regulate genes. Interestingly, Ca_V_2.1, the partner of β_4_ in cerebellar synapses, was among the genes regulated by nuclear β_4_ splice variants (Etemad et al., 2014b). These findings suggested a feedback mechanism by which accumulation of β_4b_ in the nuclei of inactive neurons represses expression of the Ca_V_2.1 channel. Upon activation nuclear export of β_4b_ relieves repression of Ca_V_2.1 and is available for incorporation of new Ca_V_2.1 channel complexes into synapses of cerebellar neurons (Etemad et al., 2014b).

A channel-independent role of β subunits in transcriptional regulation is not limited to β_4_ splice variants. Also the β_3_ subunit has also been identified as an interaction partner of a transcription factor. Upon co-expression with Pax6(S), β_3_ translocates into the nucleus of HEK cells and blocks the transcriptional activity of Pax6(S) (Zhang et al., 2010). Together these diverse functions of β subunits suggest that the high molecular diversity among the auxiliary subunits is not only important for organizing a multitude of up- and down-stream signaling pathways around the calcium channels, but also for calcium channel independent functions of auxiliary subunits.

## Non- Canonical Auxiliary Calcium Channel Subunits

### Calcium Sensor Proteins

In addition to the canonical Ca_V_ auxiliary subunits (β, α_2_δ, and γ), a number of proteins are part of calcium channel complexes and participate in functions like the regulation of channel properties or channel trafficking. Several of these non-canonical channel subunits are members of the superfamily of calcium sensor proteins (CaS). The prototypical calcium sensor protein calmodulin (CaM) governs calcium-dependent inactivation (CDI) and facilitation (CDF) of Ca_V_1 and Ca_V_2 channels. CaM is constitutively tethered to the IQ domain in the C-terminus of Ca_V_1 and Ca_V_2 channels (Halling et al., 2006). It contains two high affinity calcium binding domains (EF hands) in its N- and C-terminal lobes, and calcium binding to either one of the lobes causes conformational changes which influence CDI and CDF (Peterson et al., 1999; Van Petegem et al., 2005). Using CaM mutants impaired in calcium binding in either the N- or the C-lobe, it was demonstrated that the individual lobes control CDI and CDF in a Ca_V_ isoform-specific manner. In Ca_V_1.2 the C-lobe governs CDI, while the N-lobe controls CDF. In Ca_V_2 it is the opposite, with the N-lobe controlling CDI and the C-lobe CDF (DeMaria et al., 2001; Lee et al., 2003). In addition to controlling CDI and CDF, CaM has also been implicated in the trafficking of Ca_V_1.2 channels in neurons (Wang et al., 2007). Therefore CaM has all the characteristics of an auxiliary subunit: it is constitutively associated to the channel, it modulates its gating properties, and it is involved in the membrane expression of Ca_V_1 and Ca_V_2 channels (Lee et al., 2002; Tippens and Lee, 2007). In Ca_V_1 channels co-expression of another calcium-binding protein, CaBP1, causes suppression of CDI (Yang et al., 2006) by displacing CaM from the IQ domain and interacting with the cytoplasmic N-terminal domain of the channel (Zhou et al., 2004). In Ca_V_2.1, CaBP1 binds to a site down-stream of the IQ domain and has opposite effects. It enhances the rate of inactivation and shifts the voltage-dependence of activation to more positive potentials, with a simultaneous loss of CDF (Lee et al., 2002). Visinin-like protein 2 (VILIP2), a neuronal calcium binding protein related to CaBP1, has the opposite effects. Upon co-expression with Ca_V_2.1, VILIP2 slows down the rate of inactivation and enhances CDF (Lautermilch et al., 2005). Therefore differential expression of CaBP1 and VILIP2 at synapses yield opposite modulation of the Ca_V_2 calcium currents in response to trains of action potentials, with CaBP1 decreasing the vesicle release and VILIP2 enhancing it.

### Monomeric G-proteins

The most potent endogenous regulators of VGCCs are the members of the RGK family of Ras-related monomeric G-proteins. The RGK family was named after its four members (Rad, Rem1, Rem2 and Gem/Kir), each of which can powerfully inhibit Ca_V_1 and Ca_V_2 currents (Yang and Colecraft, 2013). Three distinct mechanisms for channel inhibition by RGK proteins have been described: (1) enhancement of dynamin-mediated endocytosis of channels, (2) reduction of the open probability (Po) without changes in voltage sensor movement, and (3) voltage-sensor immobilization (Yang and Colecraft, 2013). All RGK proteins contain a guanine nucleotide binding domain (G-domain), two regions that alter their conformation upon GTP/GDP exchange, and a C-terminus with basic and hydrophobic residues that targets RGK proteins to the plasma membrane. Notable, membrane targeting of the RGK proteins is necessary for VGCC current inhibition (Correll et al., 2008; Fan et al., 2010). All RGKs bind to all β isoforms at a region distinct from the α binding pocket (ABP), which binds to the AID in the α_1_ subunit. The affinity of the RGK/β association is an order of magnitude lower than the interaction between β and the AID (Fan et al., 2010; Xu et al., 2015). In addition to the binding sites on the β subunit, RGK-binding sites also have been suggested on the α_1_ subunits, particularly on the N-terminus (Yang and Colecraft, 2013). Using a mutated β subunit, which interacts with the α_1_ subunit but not with the RGK proteins, it was demonstrated that two of the three identified mechanisms of current inhibition by RGK, namely enhanced endocytosis and decreased P_O_, rely on β-binding of RGK proteins. In contrast, immobilization of the voltage-sensor by RGKs is independent of β-binding (Yang and Colecraft, 2013). However β subunits are absolutely required for RGK inhibition of Ca_V_ currents. Using a β subunit with a weakened affinity for the AID, which could be easily washed off from inside-out patches, it was demonstrated that in the absence of the β subunit Gem can still bind the channel, but not inhibit its current (Fan et al., 2010). The necessity of a β subunit in the VGCC complex for RGK inhibition is consistent with the notion that RGK have no effect on Ca_V_3 channels that do not contain β. The requirement of a β subunit in the VGCC complex for RGK inhibition differs from that of G_βγ_ inhibition (Fan et al., 2010). Unlike G_βγ_ inhibition, RGK inhibition does not require a rigid α-helical structure of the I-II loop. The physiological relevance of RGK proteins remains elusive, although RGK modulation of Ca_V_ channels has been implicated in diverse processes such as cell morphogenesis, migration and apoptosis. Furthermore it has been suggested that Rad may shorten QT intervals and prevent arrhythmias in heart (Yada et al., 2007). In dorsal root ganglia Ca_V_2.1-inhibition by RGK proteins may contribute to the homeostatic mechanisms, which promote plasticity and neuro-regeneration after injury (Scamps et al., 2015).

## Dynamic Subunit Composition of VGCC Complexes

So far we have described how the great molecular diversity of calcium channels is important to determine which up-stream signaling proteins can associate with and modulate the calcium current and which down-stream signaling pathways will be initiated by the calcium influx through Ca_V_ channels. If the protein–protein interactions between the channel and its various subunits and regulatory proteins are stable, changes of these modulatory mechanisms or their down-stream signaling processes could only occur at the rate of protein turnover. Altering the composition of the channel complex would require endocytosis of the existing complex and synthesis and incorporation of a new complex with different subunit composition. On the other hand, if interactions within the channel complex are dynamic, the regulation of the channel properties and the down-stream effects could be quickly changed by dynamic exchange of channel subunits and/or regulatory proteins.

In native signaling complexes of cultured neurons and skeletal myotubes the pore-forming α_1_ subunits of VGCC are stably incorporated and turn over on a minute/hour-timescale (Di Biase et al., 2011; Campiglio et al., 2013). An earlier report of depolarization-induced turnover of Ca_V_1.2 channels on a millisecond timescale could not be confirmed (Green et al., 2007). However, FRAP analysis and single particle tracking provided experimental evidence for the existence of a minor fraction of mobile Ca_V_1.2 channels in neurons (in addition to the major fraction of stable channels), providing a limited capacity for short-term adaptation (Di Biase et al., 2011). This rapid modulatory capacity could be increased if the auxiliary subunits could dynamically interact with the pore-forming α_1_ subunit. This seems to be the case for specific β and α_2_δ subunits.

The β subunit interacts with the AID, a conserved 18 amino acid peptide on the I-II loop of α_1_ (Pragnell et al., 1994). All currently available studies measured an affinity of the AID/β interaction in the low nanomolar range, suggestive of stable AID/β association. This high affinity interaction was independent of the AID/β pairs and of the method employed (De Waard et al., 1995; Geib et al., 2002; Opatowsky et al., 2003; Van Petegem et al., 2008). However, in intact cells, several studies demonstrated competition for α_1_ or β binding by peptides or proteins, indicative of a reversible α_1_/β interaction. For example application of AID peptide on inside-out patches of HEK cells expressing Ca_V_1.2/β_2a_ channel complexes resulted in a rapid decrease in the open probability, consistent with the dissociation of β_2a_ from Ca_V_1.2 and β_2a_ binding to the AID peptide (Hohaus et al., 2000). In Xenopus oocytes expressing Ca_V_2.3/β_1b_ channel complexes, injection of β_2a_ caused a decrease in the inactivation rate of the calcium current, consistent with β_1b_ in the channel complex being replaced by β_2a_ (Hidalgo et al., 2006). Finally, single channel recordings of HEK cells co-expressing Ca_V_1.2 with different ratios of β_1a_ and β_2b_ showed changes in the open probability, suggesting that distinct β subunits can sequentially associate with the same Ca_V_1.2 channel on a minute time scale (Jangsangthong et al., 2011). In a recent study we analyzed the mobility and interactions of calcium channel subunits in the context of a functional signaling complex in differentiated myotubes (Campiglio et al., 2013). Using a FRAP approach we demonstrated that the skeletal muscle channel isoforms α_1S_ and β_1a_ form a stable complex in the triads. In contrast, non-skeletal muscle β subunits (β_2a_ and β_4b_) dynamically interacted with L-type calcium channels. The observed dissociation properties of these β isoforms would enable rapid modulation of the channel by dynamic exchange of different β subunits. As of today experimental proof that channel modulation by dynamic exchange of β subunit occurs under physiological conditions is still missing. Also unresolved is the question as to how the necessary rapid change in the concentration of the β isoforms could be accomplished. However, many neurons simultaneously express multiple isoforms of auxiliary subunits, and in cerebellar neurons the activity-dependent export of nuclear β_4_ isoforms could rapidly change the relative availability of different β_4_ splice variants. Alternatively, a specific β isoform may become available after rapid post-translational modification, e.g. phosphorylation/ dephosphorylation, or after dissociation from a binding partner. A recent study suggests that BARP (β-anchoring and -regulatory protein), a transmembrane protein expressed in the brain and in pancreas, could act as such a donor of β subunits (Beguin et al., 2014).

The molecular determinants of the interaction between the α_2_δ and the α_1_ pore-forming subunit remain elusive. However it has been reported that the MIDAS motif in the VWA domain of α_2_δ-1 and α_2_δ-2 is essential for the increase in expression and the decrease of the turnover of Ca_V_1 and Ca_V_2 calcium channels in the plasma membrane (Canti et al., 2005; Hoppa et al., 2012). Although α_2_δ-1 was purified from the calcium channel complexes of skeletal muscle (Curtis and Catterall, 1984), mass spectrometry failed to detect α_2_δ as stable parts of Ca_V_2 channel complexes in brain (Muller et al., 2010). According to this study Ca_V_2 channels are part of macro-molecular signaling complexes in which they interact, directly or indirectly, with a selection of about 200 up- and down-stream signaling and scaffold proteins. Biochemically some of these interactions may be stronger than that between Ca_V_2 and its α_2_δ subunit (Muller et al., 2010). Also, upon co-expression in muscle or nerve cells, colocalization of α_2_δ subunits and VGCCs is weaker than that of β subunits (Schredelseker et al., 2005; Obermair and Flucher, 2013). Together these data suggest that α_2_δ subunits with VGCCs interact with a lower affinity than β subunits and that the interaction of α_2_δ with the channel complex may be reversible. The dynamic interaction of α_2_δ-1 with the DHPR in skeletal muscle might result in quick change of the current activation properties of the VGCC currents. siRNA-mediated depletion of α_2_δ-1 in skeletal myotubes resulted in an acceleration of Ca_V_1.1 current activation (Obermair et al., 2005). A component of fast-activating currents was also observed in normal myotubes (Obermair et al., 2005; Obermair et al., 2008). Therefore dissociation and association of α_2_δ from the DHPR complex may occur in muscle cells under physiological conditions.

A dynamic interaction of auxiliary subunits with the calcium channels can result in differential modulation of the channel properties. In the case of β subunits, the β_2a_ subunit confers to the channel slower inactivation kinetics compared to the other β isoforms (Olcese et al., 1994; Buraei and Yang, 2010). However in the case of calcium binding proteins the channel properties are modulated in opposite directions. For example in Ca_V_1 channels displacement of CaM by CaBP1 causes a suppression of CDI. In Ca_V_2.1 channels CaBP1 enhances the rate of inactivation and shifts the activation to more positive potentials, while VILIP2 slows down the inactivation and enhances CDF. Therefore Ca_V_2.1 channels that exchange CaBP1 for VILIP2 will go from a low to a high conductance regime and, thus, will increase the release probability of synaptic vesicles (Catterall, 2010).

Because the auxiliary subunits play an important role in organizing molecular machines and up- and down-stream signaling pathways their dynamic exchange might not only alter the channel properties, but also their association and functional interaction with any of these signaling pathways (Table1). For example channels associated with β_2a_ will be protected from AA inhibition whereas channels associated with β_1_, β_3_, or β_4_ are not. Ca_V_1.3 channels bound to β_1b_, but not to β_4_, will undergo CDF mediated by erbin. Or upon β-adrenergic stimulation Ca_V_1.2 channels bound to β_1b_ will be more strongly modulated channels associated with β_2a_. On contrary, Ca_V_1.3 channels bound to β_2a_ will be more strongly modulated compared to those associated with β_3_ (Liang and Tavalin, 2007). Or channels associated with β_1_ and β_2_ isoforms, but not those associated to β_3_ and β_4_, will undergo CDF mediated by CaMKII. Or presynaptic channels associated with β_3_ and β_4a_, but not with β_4b_, will bind synaptotagmin I and be located in proximity of the synaptic release machinery, whereas only channels associated to β_4b_ will be able to modulate transcriptional regulation. In addition to dynamic exchange between two isoforms of the same channel protein, channels devoid of any β subunit lose the ability to undergo RGK and G_βγ_-protein inhibition, and, in the case of Ca_V_2 channels, also to undergo PKC modulation.

Whereas the calcium channels are very stable in the plasma membrane, the auxiliary β and α_2_δ subunits can dynamically interact with the channel complex. This further increases the molecular diversity of VGCC at the membrane. Exchange of one auxiliary subunit isoform with another can result in differential modulation of the channel activity, in different protein–protein interactions, and in altered down-stream signaling.

## Conclusion

Whereas co-expression studies in heterologous cells highlighted the effects of auxiliary subunits on channel functions, in their native context the auxiliary subunits appear to be involved in orchestrating macromolecular signaling complexes involved in a multitude of calcium-regulated cell functions. The great molecular diversity endowed to VGCC by the auxiliary channel subunits and other associated proteins enables them to specifically interact with different up-stream regulators and down-stream targets localized within the confinement of the calcium micro- or nanodomain. Whereas some auxiliary subunits are stably associated with a particular VGCC signaling complex, others are capable of dynamically exchanging with VGCCs. Whether such dynamic exchange of auxiliary subunits and interacting proteins indeed differentially regulates native cell functions remains to be demonstrated. New experimental approaches and sophisticated cell systems pave the way for experiment to address this pending problem in calcium channel research.
